# Identification of Key Genes and Pathways Associated with Frailty and Exercise Effects Using a Network and Evolutionary Approach

**DOI:** 10.3390/genes16080976

**Published:** 2025-08-19

**Authors:** Kyoko Naito, Hiromichi Akahori, Yoshinori Muto, Tomoyoshi Terada

**Affiliations:** 1 United Graduate School of Drug Discovery and Medical Information Sciences, Gifu University, 1-1 Yanagido, Gifu 501-1194, Japan; naito-kyoko@chubu-gu.ac.jp; 2 Department of Nursing, Faculty of Nursing and Rehabilitation, Chubu Gakuin University, 2-1 Kirigaoka, Seki 501-3993, Japan; 3 Department of Functional Bioscience, Gifu University School of Medicine, 1-1 Yanagido, Gifu 501-1193, Japan; h.akahori@gmail.com; 4 Institute for Glyco-core Research (iGCORE), Gifu University, 1-1 Yanagido, Gifu 501-1193, Japan; muto.yoshinori.m1@f.gifu-u.ac.jp

**Keywords:** frailty, exercise, evolutionary analysis, positive selection, human accelerated region, aging genes, pathway analysis

## Abstract

Background: Frailty is an aging-associated syndrome involving a loss of physiological reserve and function, with decreased ability to recover from physical and psychosocial stress. However, the etiology and pathogenesis of frailty remain largely unknown. Aim: This study aimed to investigate key genes involved in frailty pathogenesis, exercise effects, and their contributions. Methods: We performed a weighted gene co-expression network analysis using a microarray dataset. By using the positive selection (PS), human accelerated region (HAR), and aging gene sets, we identified key genes for frailty and exercise-related genes. Results: We identified magenta and pink modules that have the most significant enrichments for the evolutionally elaborated genes. A functional enrichment analysis (FEA) revealed that genes related to redox-process regulation and extracellular-matrix organization were enriched in magenta and pink modules, respectively. We observed that six of the evolutionarily imprinted genes in the modules (*MEOX2*, *PLCB4*, *LPAR6*, *SH3KBP1*, *APP* and *SPON1*) were highly connected and showed signs of hub properties, which might play crucial roles in frailty- and exercise-related mechanisms. Conclusions: Further investigation into the functions of the identified modules and their member genes could aid in identifying diagnostic biomarkers and therapeutic targets for frailty.

## 1. Introduction

Frailty is an aging-associated syndrome defined by several different assessments as a loss of physiological reserve and function, with decreased ability to recover from physical and psychosocial stress [1,2,3]. Frail individuals are challenged by increased risks of falling, disability, hospitalization, postoperative complications, and mortality [1,4,5]. It is defined as “a state of increased vulnerability, resulting from age-associated declines in reserve and function across multiple physiologic systems, such that the ability to cope with every day or acute stressors is compromised” [1,6,7]. The most commonly used and highly regarded operational definitions are the Fried frailty phenotype [1] and the Frailty Index (FI) [8,9,10]. Specifically, the Fried frailty phenotype is defined by the presence of at least three of five criteria: unintentional weight loss (≥10 lbs in the past year), self-reported exhaustion, weakness (indicated by grip strength), slow walking speed, and low physical activity (measured as active kilocalories expended per week) [1].

The highest frailty prevalence of physical frailty, i.e., 22% of the adult population, was reported in Africa, with the corresponding prevalence in the Americas at 17%; the lowest prevalence was reported in Europe (8%) [11]. The mechanisms that underlie frailty are complex, and frailty is driven by multiple systemic changes that include inflammation, stem cell exhaustion that affects regeneration, DNA damage, changes in metabolism, and hormonal imbalances, which collectively lead to increased vulnerability and a decline in overall health and function [12,13]. Exercise, nutritional support, and reducing polypharmacy are effective interventions for frailty [14,15,16,17,18,19]. Exercise training, in particular, has been postulated as a key tool to prevent and reverse the impacts of frailty [15]. However, the effects of these approaches in the treatment of frailty have not been fully evaluated [2]. Various biomarkers and associated genes that are associated with frailty have also been studied, but it is not yet known which genes play roles in frailty, or how these genes contribute to frailty [12,20]. Numerous studies are being conducted from various perspectives, but the mechanisms underlying frailty have yet to be elucidated, and adequate treatments have yet to be established.

Comparative evolutionary analyses of human genes have identified several key disease genes and provided important insights [21]. For example, a comparative genomic study revealed that HARs (human accelerated regions) are linked to the evolution of human-specific traits, such as cognitive ability [21]. It has been speculated that (i) changes that occurred in some HARs over the course of human evolution might have provided neural-system improvements, and (ii) these changes are associated with neurological disorders [22,23,24]. An evolutionary approach that enables the use of combinations of apparently otherwise unrelated data from different disciplines can provide a more thorough understanding of complex diseases, according to a 2013 proposal by Bufill et al. [25]. Several research groups have clarified that as aging progresses, multiple causes and factors contribute to the development of frailty, including genetic and environmental factors [12,13,26,27,28,29,30].

The relationship between frailty and exercise training appears to be predominantly human, as it has rarely been observed in other species [31]. Therefore, we speculated that investigating frailty from an evolutionary perspective could significantly enhance the identification and elucidation of both the causes of frailty and effective treatments for it. We conducted a weighted gene co-expression network analysis (WGCNA) to identify frailty- and exercise-related gene modules in order to gain deeper insights into the pathogenesis of frailty. We used an evolutionary approach and referred to evolutionary data for HAR genes, positive selection (PS) genes, and aging-associated genes (i.e., ‘aging genes’) derived from genome-wide association studies (GWAS).

## 2. Materials and Methods

### 2.1. Microarray Data Acquisition and Sample Description

The microarray dataset, designated GSE117525, was retrieved from the Gene Expression Omnibus (GEO) database (https://www.ncbi.nlm.nih.gov/gds (accessed on 29 November 2022)), as originally described by Hangelbroek et al. The GSE117525 data, based on the Affymetrix Human Gene 1.1 ST Array platform, were derived from muscle biopsy samples collected from healthy young subjects (*n* = 53), healthy older subjects (*n* = 73), and frail older subjects (*n* = 61). The dataset also includes follow-up samples from healthy older subjects who completed 6 months of exercise training (*n* = 41) and from older subjects who completed 6 months of exercise training (*n* = 31). The training regimen consisted of the same progressive full-body resistance-type exercise for both the healthy older subjects and the frail older subjects. The former trained three times per week, whereas the latter trained only two [32].

### 2.2. Differentially Expressed Gene Screening

The raw signal intensities of the GSE117525 data were normalized using Chipster software ver. 3.12.3 (https://chipster.csc.fi/, accessed on 29 November 2022) to screen for differentially expressed genes (DEGs) [33]. A significance analysis of microarrays was conducted for this purpose. We calculated the fold changes (FCs) in individual gene expressions, considering DEGs significant if they had a *p*-value less than 0.05 and a |log_2_FC| value greater than 1.5. A gene expression significance analysis was performed for four pairs of subject groups: healthy young vs. healthy older subjects, healthy older vs. frail older, healthy older vs. healthy older after 6 months of exercise training, and frail older vs. frail older after 6 months of exercise training. For each gene, the gene significance (GS) was calculated as the −log_10_ of the *p*-value from Student’s *t*-test as a measure of differential expression among young subjects, healthy older subjects, frail older subjects, healthy older subjects after 6 months of exercise training, and frail older subjects after 6 months of exercise training. The module significance (MS) was also determined.

### 2.3. WGCNA

We performed a WGCNA to identify (i) co-expression modules associated with frailty and exercise training, and (ii) the key genes within these modules [34]. The WGCNA package, implemented in R, was utilized to construct an unsigned weighted gene co-expression network based on the expression values of 259 microarray datasets. We used the blockwise module function in the WGCNA package to construct this network. First, we calculated the similarity matrix for each pair of genes based on their Pearson’s correlation value. This similarity matrix was then converted into an adjacency matrix. Next, we determined the topological overlap matrix (TOM) and its corresponding dissimilarity value (1-TOM). We applied a dynamic tree cut algorithm to identify gene co-expression modules. The modules were merged using a minimum height of 0.25, which was the default setting.

The WGCNA results revealed 30 modules. To assess the relationship between each of the modules and frailty and exercise training, we calculated the correlations of the eigengenes across various frailty and exercise training samples. To identify genes significantly connected within the modules, we calculated ‘kME’.

### 2.4. Gene Annotation and Enrichment Analysis

We utilized ClueGO to detect gene ontology (GO) terms significantly associated with the modules from WGCNA [35]. “GO term analysis” was conducted to detect biological processes and molecular functions using the default settings. A *p*-value of less than 0.05 was considered significant for GO terms. Additionally, we investigated a “Reactome pathway analysis” using clusterProfiler with a strict FDR cutoff of <0.05 [36]. We constructed a bar plot using gene ratios and adjusted *p*-values to depict between-cluster differences in enriched functional categories.

### 2.5. Construction of a Human PPI Network

Protein interaction data were downloaded from the “iRefIndex database” for the initial dataset. This database integrates several primary PPI databases [37]. To enhance the confidence and completeness of the PPI network, we incorporated PPIs from the extensive “BioPlex 2.0 interaction dataset” [38]. We included interaction data that extended beyond direct physical bindings from previous research [39]. We constructed a human PPI network comprising 22,616 nodes and 515,015 edges.

### 2.6. PPI Subnetworks Among Proteins Encoded by Frailty- and Exercise-Associated Genes

The genes from the magenta and pink modules were mapped to the human PPI network, and the maximal connected components were extracted as protein interaction subnetworks using “Cytoscape ver. 3.8.2” [40]. We employed the “molecular complex detection (MCODE) clustering tool” with default settings [41] to analyze the resulting network and identify highly interconnected PPI clusters. “CytoHubba” was used to assess hub genes in the PPI networks [42]. We calculated potential hub genes based on the following metrics: “node degree, maximal clique centrality (MCC), betweenness centrality, and maximum neighborhood component (MNC)” [42,43].

### 2.7. Enrichment Analysis of HAR, PS, and Aging Genes

The HAR genes were retrieved from a study by Doan et al. [22], which describes a comparative genome analysis identifying conserved genomic loci with elevated divergence in humans. A total of 2737 HARs were identified, representing 2163 HAR-associated genes. The lists of genomic regions showing PS signatures from the PopHumanScan were downloaded [44]. In this study, we included only positively selected regions that overlapped with protein-coding sequences as PS genes. Aging-associated genes (aging genes) were obtained from the GWAS Catalog [45] and the DisGeNET database [46]. We conducted an enrichment analysis using the three sets of PS, HAR, and aging genes, employing a one-sided Fisher’s exact test.

## 3. Results

### 3.1. WGCNA Results

**Figure 1:**
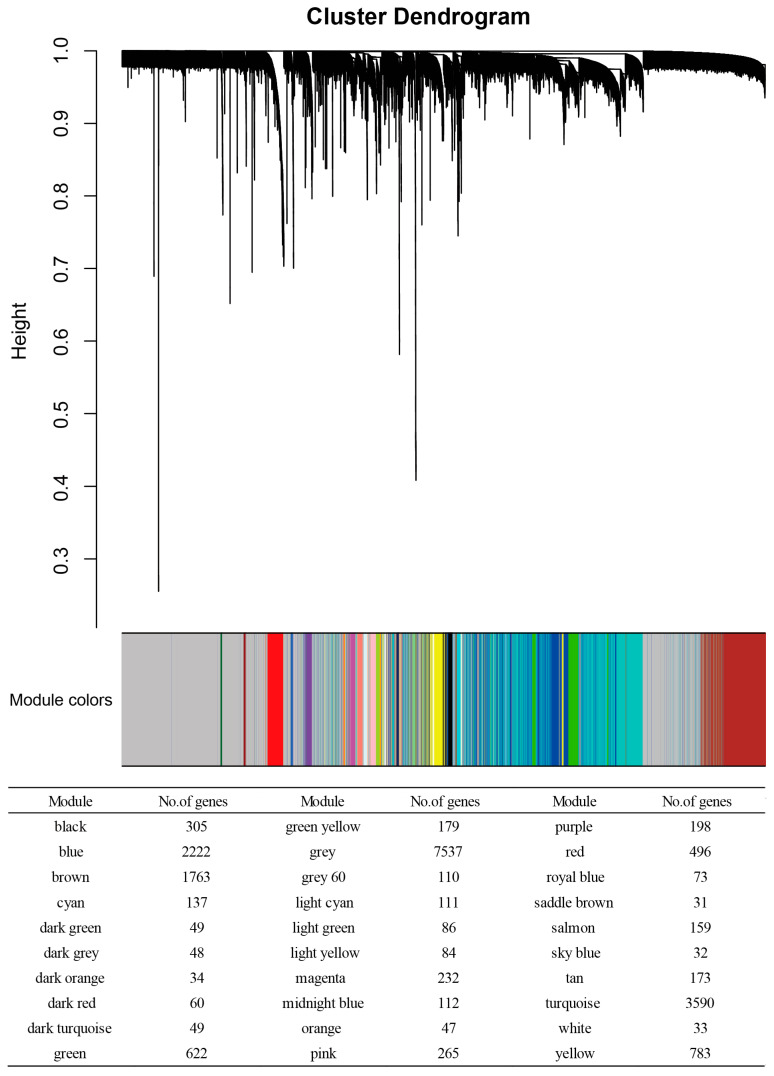
Results of the WGCNA of the transcriptome in young, healthy older, frail older, healthy older after 6 months of exercise training, and frail older after 6 months of exercise training. Gene clustering is visualized as a dendrogram, with modules indicated by distinct colors. Thirty modules were identified.

After the data preprocessing, we obtained 19,620 genes’ expression matrices from the 259 samples. The genes with similar expression patterns were then grouped into modules with the use of the R program’s WGCNA package. We identified 30 modules by following the protocol described above in Section 2.3. Each module’s number of genes is presented in Figure 1. Two additional network metrics were used to identify aging, frailty and training exercise-associated modules: “GS values” and “MS values”.

**Figure 2:**
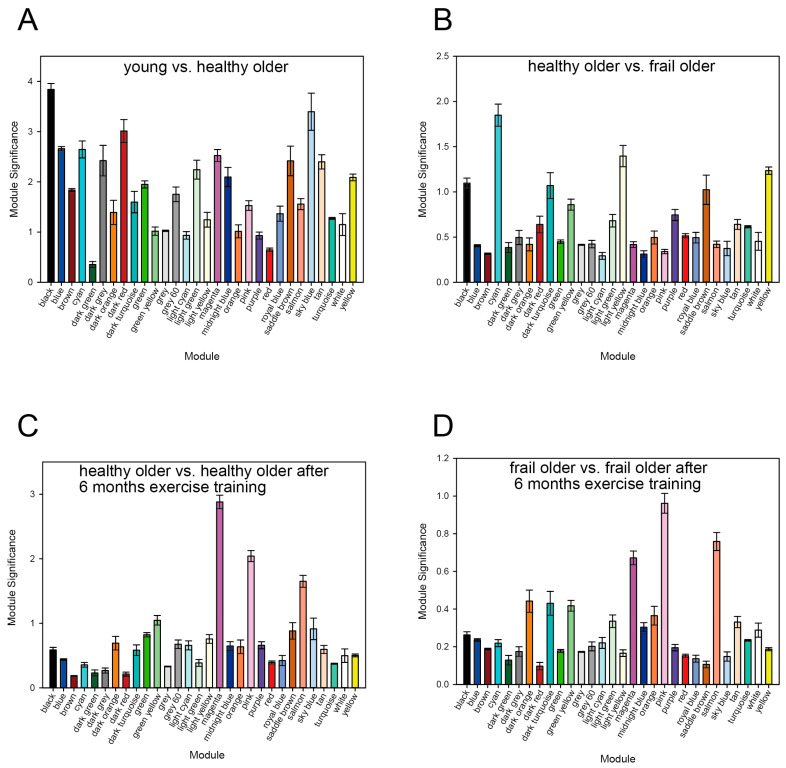
Module significance (MS) values. The black, sky-blue, and dark-red modules were highly enriched with DEGs in the young vs. healthy older comparison (**A**). The cyan, light yellow, and yellow modules were highly enriched in the healthy older vs. frail older comparison (**B**). The magenta, pink, and salmon modules were highly enriched in the healthy older group before vs. healthy older group after 6 months of exercise training (**C**), and in the frail older group before vs. after 6 months of training (**D**). Bars represent mean values ± standard error of the mean (SEM).

As shown in Figure 2, the top three modules with the highest MS values are black, sky blue, and dark red for the healthy young vs. healthy older subject pair (Figure 2A), cyan, light yellow, and yellow for the healthy older vs. frail older subject pair (Figure 2B), magenta, pink, and salmon for the healthy older vs. healthy older after 6 months’ exercise training subject pair (Figure 2C), and pink, salmon, and magenta for the frail older vs. frail older after 6 months’ exercise training subject pair (Figure 2D). These modules were enriched with differentially expressed genes between their respective subject groups, as indicated by the increased MS values. This strongly suggests that the black, sky-blue, and dark-red modules are related to aging; cyan, light-yellow, and yellow modules are related to frailty; and the magenta, pink, and salmon modules are related to exercise training. We characterized these nine modules and evaluated gene co-expressions for further analysis.

**Figure 3:**
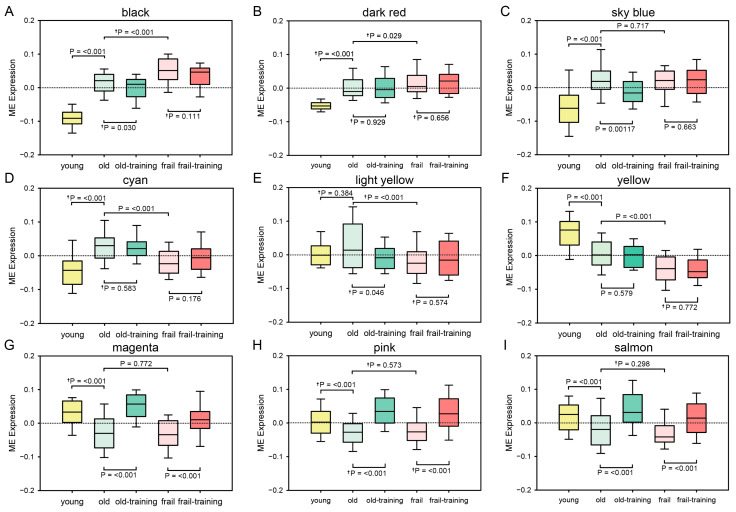
Module eigengene (ME) expression levels of the black (**A**), sky-blue (**B**), dark-red (**C**), cyan (**D**), light-yellow (**E**), yellow (**F**), magenta (**G**), pink (**H**), and salmon (**I**) modules. Columns: mean values. Bars: SEM. † Mann–Whitney U-test.

We determined the mean ME values for each module and compared individual subject groups using Student’s *t*-test. The Mann–Whitney U-test was applied for variables that were not normally distributed. Figure 3 shows the mean ME value of each module for five subject groups. The mean ME values of the black, sky-blue, and dark-red modules were significantly greater in the healthy older subjects compared to the young subjects (Figure 3A–C). The mean ME values of the cyan, light-yellow, and yellow modules were significantly increased in the healthy older subjects in comparison to the frail older subjects (Figure 3D–F). The mean ME values of the magenta, pink, and salmon modules were significantly greater in the subjects after six months of exercise training compared to the non-training control subjects (Figure 3G–I).

### 3.2. Enrichment of HAR, PS, and Aging Genes in Each Module

The functional significance and properties of a gene often reflect the gene’s evolutionary history. For example, HARs are human genome regions that have evolved over the past 5–6 million years after humans and chimpanzees diverged from their last common ancestor [47]. Comparative genomic studies revealed that several HAR-associated genes are linked to a variety of human-specific diseases, e.g., preeclampsia and autism-spectrum disorder and human traits [22,48,49]. PS genes, as deduced from human polymorphism data, have also been shown to be shaped by recent evolutionary forces and to be linked to human specific traits [50].

**Figure 4:**
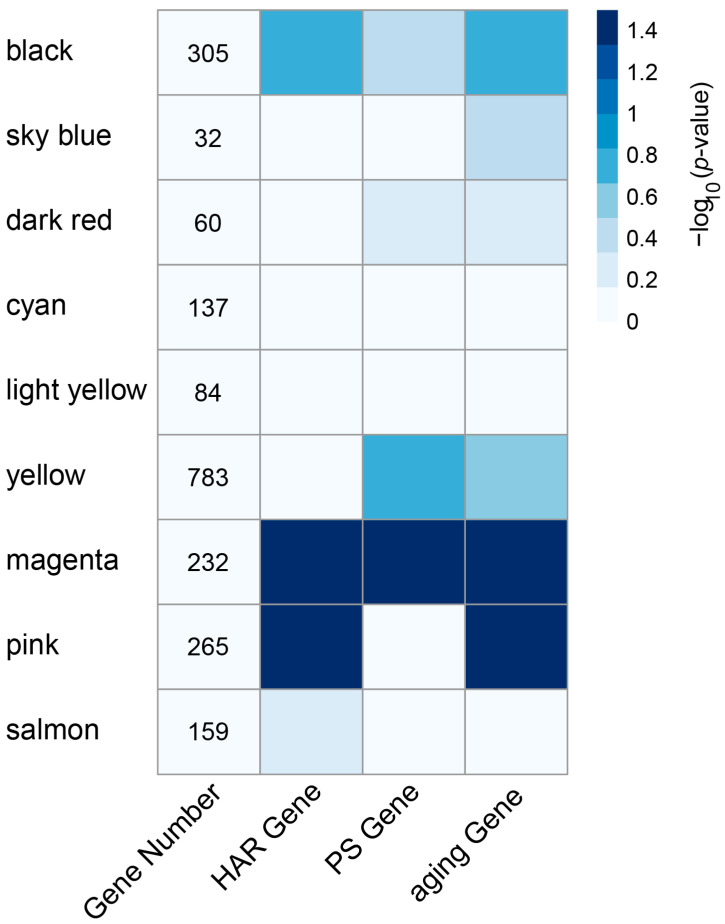
Enrichment analysis of HAR, PS, and aging genes in each module. Module-level enrichment was assessed for two evolutionary gene sets—PS genes and HAR genes—as well as for the aging gene set. Fisher’s exact test was used for statistical evaluation. Color intensity in each cell reflects the −log_10_ (*p*-value) of enrichment significance.

We retrieved PS and HAR genes from earlier investigations [22,51] to conduct a search in the modules identified in the present study, and these PS and HAR gene sets were used to assess the top MS modules’ enrichment. We also used an aging gene set to evaluate the enrichment of the modules. As shown in Figure 4, the magenta and pink modules showed a strong tendency to contain HAR genes and aging genes. Of the nine top MS modules, significant enrichment of the HAR and aging gene sets was observed in the magenta and pink modules, respectively. In contrast, significant enrichment for the PS gene set was only observed in the magenta module (*p* < 0.05, Fisher’s exact test). The black, sky-blue, dark-red, cyan, light-yellow, yellow, and salmon modules were not significantly enriched for the HAR, PS and aging gene datasets. It is notable that magenta and pink modules are the most significant modules related to exercise training (Figure 2C,D), and both module member genes are highly expressed in the subjects after 6 months of exercise training (Figure 3G,H). These data suggest that the magenta and pink modules are potentially important for frailty-exercise training research from an evolutionary perspective, and we thus focused on the magenta and pink modules in our subsequent studies.

### 3.3. GO Analysis

**Figure 5:**
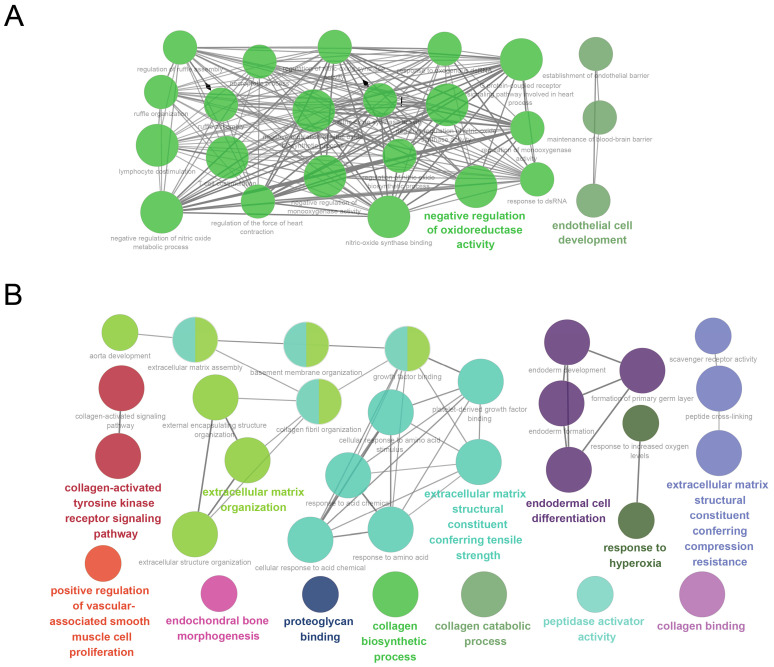
Gene annotation enrichment analysis results for the “biological process” and “molecular function” GO categories in the magenta (**A**) and pink (**B**) modules. The functionally grouped network is shown, with terms presented as nodes linked based on their “kappa scores” (threshold = 0.4).

To clarify the specific biological relevance of the magenta and pink modules, we subjected 232 genes in the magenta module and 265 genes in the pink module to GO analyses. These results indicate that the magenta module is associated mostly with negative regulation of oxidoreductase activity, regulation of nitric-oxide synthase activity, and endothelial cell development (Figure 5A). The pink module is associated mostly with the extracellular matrix structural constituent conferring tensile strength, extracellular matrix organization, endodermal cell differentiation, and the extracellular matrix structural constituent conferring compression resistance (Figure 5B). It is noteworthy that, despite the evident relationship of the magenta and pink modules with exercise training (Figure 2C,D), the “biological processes and molecular functions” that are enriched in each module are essentially different. This observation indicates that the effects of exercise differ between individuals with frailty and those who are healthy, suggesting that different genes are involved in the processes. All significantly enriched GO terms and their associated genes are shown in Supplementary Table S1.

### 3.4. PPI Analysis and the Identification of Densely Connected Clusters

**Figure 6:**
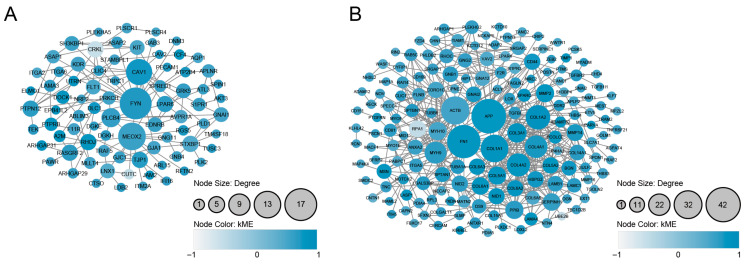
PPI networks constructed from genes in the magenta (**A**) and pink (**B**) modules. The magenta module network contains 80 nodes and 129 edges, while the pink module network contains 165 nodes and 517 edges. Node size corresponds to degree centrality, and node color intensity reflects module membership (kME values).

We mapped the 232 and 265 genes in the magenta and pink modules, respectively, to the human PPI network. The genes of the obtained PPI in the magenta module contained 80 nodes and 129 edges, and the genes of the pink module’s obtained PPI contained 165 nodes and 517 edges (Figure 6A,B). It is known that specific function- and disease-associated genes tend to interact and function together in the same biological cluster within a molecular interaction network [52]. We thus speculated that detecting such interaction clusters could contribute to the identification of both key pathways and frailty- and exercise training-associated genes, and we suspected that doing so could clarify the molecular mechanisms underlying exercise training’s effects and the etiology.

MCODE analysis revealed three densely connected clusters (C1–C3) with 8–54 genes in the magenta module and four densely connected clusters (C1–C4) with 3–46 genes in the pink module (Supplementary Table S2).

### 3.5. Enrichment of HAR, PS, and Aging Genes in the PPI Clusters

**Figure 7:**
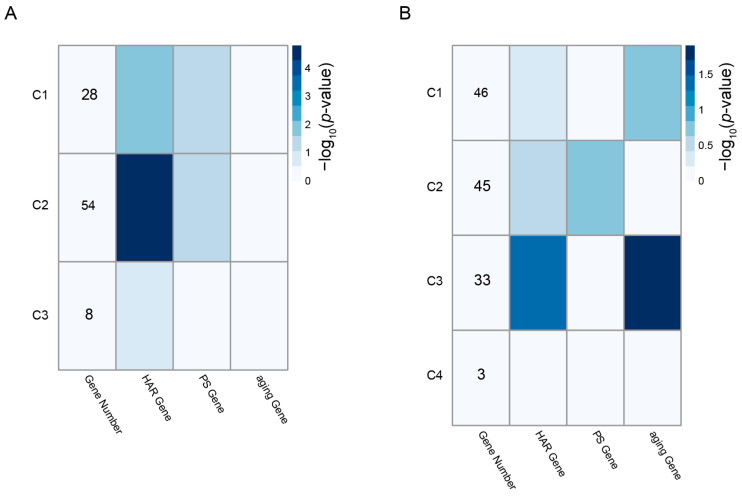
Densely connected clusters identified within the PPI networks of the magenta (**A**) and pink (**B**) modules. Three significant clusters were extracted from the magenta module and four from the pink module. Cluster-level enrichment for HAR, PS, and aging-related genes was assessed using Fisher’s exact test. Color intensity in each cell indicates the −log_10_ (*p*-value) of enrichment significance.

**Figure 8:**
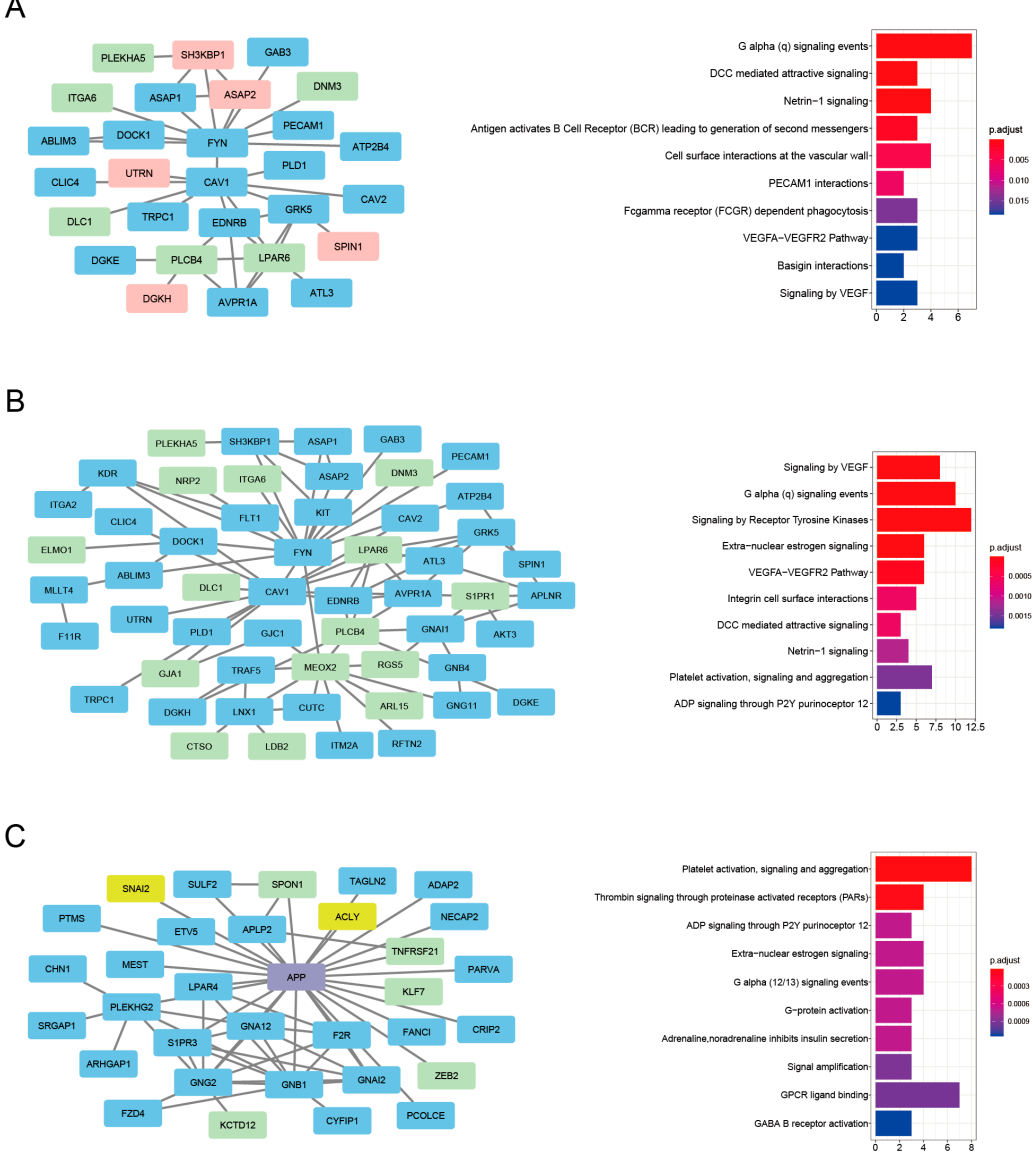
PPI clusters enriched with HAR, PS, and aging genes. (**A**) Cluster C1 from the magenta module shows enrichment for HAR and PS genes. “Functional enrichment analysis (FEA)” results for member genes are shown. (**B**) Cluster C2 from the magenta module shows enrichment for HAR genes. (**C**) Cluster C3 from the pink module shows enrichment for HAR and aging genes. HAR genes: light green. PS genes: light red. Aging genes: yellow. Genes related to both HAR and aging: purple. Bar plots display the top 10 enriched pathways, with bar length indicating gene count and color indicating statistical significance. The Benjamini–Hochberg method was used to adjust the *p*-values.

The magenta-associated cluster C1 demonstrated a significant tendency to contain HAR and PS genes (*p* < 0.05), while the magenta-associated cluster C2 was most significantly enriched for HAR genes (*p* < 0.0001) (Figure 7A). The pink-associated cluster C3 was significantly enriched for HAR genes and also for aging genes (*p* < 0.05) (Figure 7B). Figure 8 depicts the PPI network representation of these significant clusters, and the specific genes are indicated by the node color (HAR genes: light green, PS genes: pale red, aging genes: yellow, and purple for both HAR and aging genes). All data for the magenta and pink cluster genes that overlap with the HARs, PS, and aging genes are displayed in Supplementary Table S3.

To identify the specific biological relevance of the PPI clusters that are highly enriched with evolution-related genes, 28 genes in the magenta-associated cluster C1, 54 genes in cluster C2, and 33 genes in the pink-associated cluster C3 were subjected to reactome pathway enrichment analyses. These results indicated that the magenta-associated cluster C1 is associated primarily with aspects of signaling by GPCR, such as G alpha (q) signaling events. This cluster is also associated with axon guidance such as DCC-mediated attractive signaling and Netrin-1 signaling, plus signaling by the B-cell receptor such as the antigen-activated B-cell receptor (Figure 8A).

Regarding the magenta-associated cluster C2, the significant reactome pathways were associated primarily with aspects of signaling by receptor tyrosine kinase, such as signaling by VEGF and the VEGFA–VEGFR2 pathway (Figure 8B). The pink-associated cluster C3 was significantly enriched for genes involved in platelet activation, signaling, and aggregation, such as thrombin signaling through proteinase-activated receptor and ADP signaling through P2Y purinoceptor 12 (Figure 8C). The full list is provided in Supplementary Table S4.

To identify high-significance genes with key roles in the PPI networks, we applied the Cytohubba to a hub gene analysis [42]. Table 1 shows the highly connected HAR, PS, and aging genes (including their full names) in the PPI network clusters. As can be seen from the node-degree distribution, *MEOX2*, *PLCB4*, *LPAR6*, and *SH3KBP1* in the magenta-associated C1 and C2 clusters were the most highly connected in the PPI networks. In addition, among the nodes in the pink-associated cluster C3, *APP* and *SPON1* were detected as the most connected nodes overlapping with the HAR gene set. The values of the hub gene parameter betweenness centrality were high in these high-node-degree genes (Table 1). Taken together, these results indicate that these highly connected hub genes—which are possible key genes that have been evolutionarily elaborated for regulating exercise training and frailty—may critically affect the pathways that are associated with the magenta and pink clusters.

## 4. Discussion

The WGCNA performed herein to identify frailty–exercise co-expression modules in publicly available gene expression datasets revealed 30 modules that are highly enriched for genes which are differentially co-expressed in various subject groups. Using the PS and HAR gene sets, we performed an enrichment analysis to evaluate the signatures of evolutionary forces that affect the WGCNA-detected modules. Earlier studies indicated that HAR genes’ divergence between humans and other primates reflects these genes’ potential roles in human trait (e.g., cognitive ability) evolution [21,22]. PS genes that were inferred with the use of human polymorphism data have been described as reflecting the recent adaptations of humans to various new environments [53]. The present study obtained evidence that both PS and HAR genes are significantly overrepresented in the exercise training-related (i.e., magenta and pink) modules. Since the magenta and pink module genes were highly expressed in the subjects after six months of exercise training, we speculate that evolutionary forces might preferentially affect genes that are related to human training and frailty-recovering process. The magenta and pink modules identified in our study will be useful for investigations of the molecular mechanisms of frailty and exercise training effects.

Our GO term enrichment analysis of the magenta and pink exercise-associated co-expression modules revealed that the magenta module is enriched for genes associated with the negative regulation of oxidoreductase activity and endothelial cell development. An integral part of human exercise-associated metabolism appears to be redox processes. Although reactive species had traditionally been thought to be exclusively detrimental molecules, accumulating evidence has indicated that exercise-induced reactive species are critical upstream signals for (i) activating redox-sensitive transcription factors, and (ii) inducing the expressions of genes that are associated with exercise [54,55,56]. In contrast, the pink module exhibited an enrichment of genes associated with the extracellular matrix structural constituent conferring tensile strength, extracellular matrix organization, and endodermal cell differentiation. It has been demonstrated that essentially all animal cells are connected through the extracellular matrix (ECM), which is a network-like scaffold [57]. The skeletal–muscle ECM is critical for the production of muscle force and for regulating several physiological processes during growth, regeneration and remodeling; assessments of ECM remodeling can, therefore, provide useful information about injury, disease, and the effectiveness of rehabilitation [58]. Interestingly, although the magenta module was the most significant in the comparison of healthy older subjects versus healthy older subjects after 6 months exercise training, the pink module was the most significant when comparing frail older versus frail older after 6 months exercise training (Figure 2C,D). Our findings thus indicate that the genes and functions that are affected by exercise training are quite different between healthy older subjects and frail older subjects. Given that the GO terms and associated genes of the pink module were most strongly implicated in frail older individuals, this observation may suggest an important role for pink module genes in the transition process.

Since it could be useful to use small PPI clusters that may contain proteins that participate in similar biological processes when seeking to deduce frailty–exercise functional pathways, we used the “MCODE” clustering tool to extract protein clusters in the magenta and pink modules’ PPI network that are densely connected. A total of seven significant PPI clusters consisting of highly connected protein members were identified. Based on the significant enrichment of the HAR, PS, and aging gene datasets, the top three network clusters were obtained: the magenta-associated cluster C1, the magenta-associated cluster C2, and the pink-associated cluster C3 (Figure 8). In these PPI clusters, hub genes are highly connected to other genes, making them biologically important [59]. As shown in evolutionary modeling of gene networks, changes in a small number of hub genes can significantly impact the function of a PPI cluster [60]. Therefore, we focused on the hub genes with evolutionary footprints.

*MEOX2*, *PLCB4*, and *LPAR6* are HAR genes that were detected in the magenta-associated C1 and/or C2 clusters, and they had higher node degrees in the PPI network. *SH3KBP1* is a PS gene that was detected in the magenta-associated cluster C1, and it was also revealed that the node degree was higher in the PPI network. MEOX2 (mesenchyme homeobox 2) is unique among the nuclear transcription inhibitors [61,62,63]. GWAS identified rare copy number variants in the MEOX2 gene that are associated with early and severe phenotypes of Alzheimer’s disease [64]. *MEOX2* also controls muscle size and muscle fiber metabolism [65]. *PLCB4* (phospholipase C β4) is highly expressed in the cerebellum [66]. PLCB4^−/−^ mice showed ataxia [67]. Chen et al. revealed that PLCB4+ Purkinje neurons play an important role in associative learning [68]. LPAR6 (lysophosphatidic acid receptor 6) is the G protein-coupled receptor of the LPA family [69], and it has been known to be associated with cancer types such as hepatocellular carcinoma [70], pancreatic cancer [71] and prostate cancer [72]. SH3KBP1 (SH3-domain kinase binding protein 1) contributes to T cell-independent immune responses by linking the B-cell receptor to IKK-β/NF-κB activation [73]. APP, i.e., amyloid-β (Aβ) precursor protein, is a HAR gene (and an aging gene) that we detected in the pink module-associated cluster C3; it was revealed that the node degree was higher in the PPI network. APP is a ubiquitous membrane protein that has been observed to be associated with Alzheimer’s disease and with cerebral amyloid angiopathy; the products of APP cleavage have a variety of physiological roles that are important in neuronal development and neuronal function [74]. *SPON1* is a HAR gene detected in the pink module-associated cluster C3. It is known to bind to the BACE1 cleavage site of APP and to block the initiation of amyloidogenesis [75]. Maltais et al. revealed that Aβ deposition in the putamen is associated with the varying severity of frailty in older adults [76]. Exercise modulates the turnover of Aβ, inflammation, neurotrophin synthesis and release, and cerebral blood flow (CBF) [77]. Collectively, these findings support the possibility that PS and HAR genes are key players in the PPI clusters associated with the exercise training-related magenta and pink modules.

To date, many genes have been proposed as candidate genes for frailty [12,20,78,79], but the details remain unclear. Recently, Lin et al. [78] focused on 14 genetic loci associated with frailty index susceptibility, identifying *HTT* and *LRPPRC* as novel susceptibility genes linked to frailty risk. They found that *HTT* may increase the risk of developing frailty, whereas *LRPPRC* may offer protection against its onset [78]. Perez et al. revealed that *CDKN1A* was expressed in a small population of nuclei present only in aged samples [79]. Although PS and HAR genes appear to have conferred fitness advantages throughout human evolutionary history [22,80], it is also speculated that these genes may increase the risk of human-specific disorders through antagonistic pleiotropy [81,82]. In particular, the *MEOX2*, *PLCB4*, *LPAR6*, *SH3KBP1*, and *SPON1* genes, which we newly identified using an evolutionary approach, have been shown to play significant roles, as mentioned above. Furthermore, these genes have not previously been recognized as candidate genes for frailty. A detailed investigation of these genes may enhance our understanding of frailty treatment and the mechanisms underlying exercise effects. However, our study has several limitations. Further experimental research is needed to confirm the roles of these genes and molecular pathways. In addition, the exercise training protocols differed slightly between healthy and frail older subjects, who trained three and two times per week, respectively. Therefore, direct comparisons between these groups were not possible.

## 5. Conclusions

We performed a system-level analysis of frailty- and exercise-related gene clusters derived from WGCNA and PPI analyses, describing the characteristics of evolutionarily imprinted PS and HAR genes within these clusters. Given that the relationship between frailty and exercise training appears to be a human-specific feature, the PS and HAR genes identified in the magenta- and pink-associated clusters may play pivotal roles in exercise training processes and potentially in the pathology of frailty itself. In particular, the six PS and HAR genes (*MEOX2*, *PLCB4*, *LPAR6*, *SH3KBP1*, *APP*, and *SPON1*) in the magenta and pink modules exhibit hub properties and may serve as useful diagnostic biomarkers and potential targets for therapeutic interventions.

## Figures and Tables

**Table 1:** Human accelerated region (HAR) genes, positive selection (PS) genes, and aging genes detected in the magenta module’s C1 and C2 clusters and the pink module’s C3 cluster.

Scheme	Gene Full Name	Module	Cluster	Degree	Betweenness	HAR Gene	PS Gene	Aging Gene
PLCB4	Phospholipase C Beta 4	magenta	cluster1,2	8	675.80101	○		
LPAR6	Lysophosphatidic Acid Receptor 6	magenta	cluster1,2	6	189.06032	○		
SH3KBP1	SH3 Domain Containing Kinase Binding Protein 1	magenta	cluster1	5	161.16667		○	
MEOX2	Mesenchyme Homeobox 2	magenta	cluster2	14	2686.26898	○		
APP	Amyloid Beta Precursor Protein	pink	cluster3	42	9368.80272	○		○
SPON1	Spondin 1	pink	cluster3	3	2.4	○		

## Data Availability

The data reported in this study are available in the Supplementary Information.

## Supplementary Materials

The following supporting information can be downloaded at: https://www.mdpi.com/article/10.3390/genes16080976/s1, Table S1: List of significant gene ontology (GO) terms for the magenta and pink modules identified by the weighted gene co-expression network analysis (WGCNA); Table S2: The complete results of the molecular complex detection (MCODE) analysis for the magenta and pink modules; Table S3: Magenta and pink cluster genes overlapping with the human accelerated region (HAR) gene set, positive selection (PS) gene set, and aging gene set; Table S4: The significantly enriched reactome pathways in the magenta C1 and C2 and pink C3 protein-protein interaction (PPI) clusters and the pathways’ associated proteins; Table S5: The results of the Cytohubba analysis of the magenta and pink PPI clusters.
